# Supplementary material for: Platelet rich plasma versus placebo for the management of Achilles tendinopathy: protocol for the UK study of Achilles tendinopathy management (ATM) multi-centre randomised trial
Source: BMJ Open. 2020 Feb 12;10(2):e034076. doi: 10.1136/bmjopen-2019-034076 (PMC7044811; doi:10.1136/bmjopen-2019-034076)
Supplement: Supplementary data [file bmjopen-2019-034076supp001.pdf]

List of site codes and open date

Site	Site code	Open Date
University Hospitals Coventry and Warwickshire	UHCW	27/04/2016
The Princess Royal Hospital, Shrewsbury and Telford**	SHTL	10/06/2016
Ninewells Hospital, Dundee	DUND	15/06/2016
Norfolk and Norwich University Hospitals***	NNUH	12/08/2016
University Hospitals Leicester	LEIC	20/07/2016
Northern General Hospital, Sheffield	NGH	26/10/2016
Northumbria Hospital	NORT	04/01/2017
Leighton Hospital*	MCHT	18/01/2017
Morriston Hospital, Swansea	SWAN	04/04/2017
Arrowe Park Hospital	ARRW	28/04/2017
Wexham Park Hospital	WEX	27/06/2017
Royal Liverpool Hospital****	ROYL	17/07/2017
Robert Jones and Agnes Hunt Orthopaedic Hospital	RJAH	22/08/2017
Doncaster and Bassetlaw Hospital	DONC	08/06/2017
Royal Devon and Exeter Hospital	EXET	10/01/2018
Musgrove Park Hospital	MUSG	15/01/2018
Merthyr Tydfil	MERT	13/02/2018
Basildon University Hospital	BASL	05/03/2018
George Eliot Hospital	GEH	02/05/2018
North Tees and Hartlepool Hospital	TEES	19/06/2018
Cardiff and Vale Orthopaedic Centre, Llandough Hospital, Cardiff	CARD	03/07/2018
Alexandra Hospital, Redditch	WAH	26/11/2018
Wharfedale Hospital, Leeds Community Healthcare	LCH	29/11/2018
Imperial College London	IMPC	19/02/2019

*Closed to *recruitment August 2017*

***Closed to recruitment September 2018*

****Closed to recruitment May 2018*

*****Closed to recruitment November 2018*

Kearney RS, *et al. BMJ Open* 2020; 10:e034076. doi: 10.1136/bmjopen-2019-034076
