# Supplementary material for: Platelet rich plasma versus placebo for the management of Achilles tendinopathy: protocol for the UK study of Achilles tendinopathy management (ATM) multi-centre randomised trial
Source: BMJ Open. 2020 Feb 12;10(2):e034076. doi: 10.1136/bmjopen-2019-034076 (PMC7044811; doi:10.1136/bmjopen-2019-034076)
Supplement: Supplementary data [file bmjopen-2019-034076supp002.pdf]

Schedule of enrolment, interventions and assessments.

**Visit Window** (No. Weeks ± No. Days)	**Baseline**	**2 wk post randomisation:** telephone follow up	**3 m (± 1 m) after randomisation: postal follow up**	**6 m (± 1m) after randomisation: postal follow up**
Eligibility screen	✓			
Written informed consent	✓			
Baseline data Form	✓			
Randomisation	✓			
Intervention delivery	✓			
Pain score	✓	✓	✓	✓
VISA-A	✓		✓	✓
EQ5D-5L	✓		✓	✓
Adverse events	✓	✓	✓	✓
