# Supplementary material for: Platelet rich plasma versus placebo for the management of Achilles tendinopathy: protocol for the UK study of Achilles tendinopathy management (ATM) multi-centre randomised trial
Source: BMJ Open. 2020 Feb 12;10(2):e034076. doi: 10.1136/bmjopen-2019-034076 (PMC7044811; doi:10.1136/bmjopen-2019-034076)
Supplement: Supplementary data [file bmjopen-2019-034076supp003.pdf]

CONSENT FORM

ATM

Achilles Tendinopathy Management (ATM): A multi-centre placebo controlled randomised controlled trial comparing Platelet Rich Plasma (PRP) to placebo (imitation) injection in adults with Achilles tendon pain.

Chief Investigator: Dr. Rebecca Kearney

Site ID ☐☐☐ **Participant ID** ☐☐☐

Please INITIAL boxes do not tick

1. I confirm that I have read and understood the information sheet (version 6.1 dated 04-JAN-2019) for the above study. I have had the opportunity to consider the information, ask questions and have had these answered satisfactorily. ☐
2. I understand that my participation is voluntary and that I am free to withdraw at any time, without giving any reason, without my medical care or legal rights being affected. ☐
3. I understand that relevant sections of any of my medical notes and data collected during the study may be looked at by responsible individuals from regulatory authorities or from the NHS Trust, where it is relevant to my taking part in this research. I give permission for these individuals to have access to my records. ☐
4. I understand that appropriate personal identifying information will be collected, stored and used by Warwick Clinical Trials Unit to enable follow up of my health status. This will include the contact details for my next of kin. I understand that any information will be treated with the strictest security and confidentiality. ☐
5. I understand that the information held and maintained by The Health and Social Care Information Centre and other central UK NHS bodies may be used to help contact me or provide information about my health status ☐
6. I agree to have a blood sample taken for the purposes of the above study and that part of this sample will be used in my treatment if I am allocated to the Platelet Rich Plasma (PRP) Injection group. I understand that if I am not allocated to this group, my sample will be destroyed and not used as part of my treatment. ☐
7. I agree that I will complete questionnaires regarding the impact of the treatment on my pain, function and activity and overall quality of life. ☐
8. I agree to my GP being informed of my participation in the study. ☐
9. I agree to being sent text messages to remind me that my questionnaire is due. ☐
10. I agree to take part in the above study. ☐

Full name of patient	Signature	Date (dd/mmm/yyyy)
Full name of person taking consent	Signature	Date (dd/mmm/yyyy)

ATM CONSENT FORM v4.0 | 23-MAR-2016 Page **1** of **1**

Original copy to be kept in in the ATM site file, one copy to be kept in the patients' medical notes, and one copy to the patient.
