# Supplementary material for: Platelet rich plasma versus placebo for the management of Achilles tendinopathy: protocol for the UK study of Achilles tendinopathy management (ATM) multi-centre randomised trial
Source: BMJ Open. 2020 Feb 12;10(2):e034076. doi: 10.1136/bmjopen-2019-034076 (PMC7044811; doi:10.1136/bmjopen-2019-034076)
Supplement: Author's manuscript [file bmjopen-2019-034076.draft_revisions.pdf]

BMJ Open is committed to open peer review. As part of this commitment we make the peer review history of every article we publish publicly available.

When an article is published we post the peer reviewers' comments and the authors' responses online. We also post the versions of the paper that were used during peer review. These are the versions that the peer review comments apply to.

The versions of the paper that follow are the versions that were submitted during the peer review process. They are not the versions of record or the final published versions. They should not be cited or distributed as the published version of this manuscript.

BMJ Open is an open access journal and the full, final, typeset and author-corrected version of record of the manuscript is available on our site with no access controls, subscription charges or pay-per-view fees (http://bmjopen.bmj.com).

If you have any questions on BMJ Open's open peer review process please email info.bmjopen@bmj.com

BMJ Open

Platelet Rich Plasma versus Placebo for the management of Achilles Tendinopathy: Protocol for the UK study of Achilles Tendinopathy Management (ATM) multi-centre randomised trial

Journal:	*BMJ Open*
Manuscript ID	bmjopen-2019-034076
Article Type:	Protocol
Date Submitted by the Author:	04-Sep-2019

Complete List of Authors:	Kearney, Rebecca; University of Warwick, Warwick Clinical Trials Unit<br>Parsons, Nicholas; University of Warwick, Warwick Medical School<br>Ji, Chen; University of Warwick, Warwick Clinical Trials Unit<br>Warwick, Jane; University of Warwick, Warwick Clinical Trials Unit<br>Brown, Jaclyn; University of Warwick, Warwick Clinical Trials Unit<br>Young, Jonathan; University Hospitals Coventry and Warwickshire NHS Trust<br>Costa, Matthew; University of Oxford, Nuffield Department of Orthopaedics, Rheumatology, and Musculoskeletal Sciences

Keywords:	Achilles tendon, Tendinopathy, Injection, Platlet Rich Plasma

SCHOLARONE™
Manuscripts

TITLE PAGE

Title

Platelet Rich Plasma versus Placebo for the management of Achilles Tendinopathy: Protocol for the UK study of Achilles Tendinopathy Management (ATM) multi-centre randomised trial

Corresponding Author

Rebecca S Kearney: Associate Professor, University of Warwick, Clinical Trials Unit, Gibbet Hill Road, Coventry, CV4 7AL; R.S.Kearney@warwick.ac.uk; 02476 573156

Authors

- Nick Parsons: Associate Professor, University of Warwick, Warwick Medical School, Gibbet Hill Road, Coventry, CV4 7AL (Nick.Parsons@warwick.ac.uk)
- Chen Ji: Senior Research Fellow, University of Warwick, Clinical Trials Unit, Gibbet Hill Road, Coventry, CV4 7AL (C.J.3@warwick.ac.uk)
- Jane Warwick: Professor, University of Warwick, Clinical Trials Unit, Gibbet Hill Road, Coventry, CV4 7AL (J.Warwick@warwick.ac.uk)
- Jaclyn Brown: Senior Project Manager, University of Warwick, Clinical Trials Unit, Gibbet Hill Road, Coventry, CV4 7AL (J.Brown.10@warwick.ac.uk)
- Jonathan Young: Consultant Orthopaedic Surgeon, University Hospitals Coventry & Warwickshire NHS Trust, Trauma and Orthopaedics, Clifford Bridge Road, Coventry, CV2 2DX (Email: jonathan.young3@uhcw.nhs.uk)
- Matthew Costa: Professor Orthopaedic Trauma, Trauma Unit, Kadoorie Centre, John Radcliffe Hospital, Oxford, OX3 9DU, UK (matthew.costa@ndorms.ox.ac.uk)

Word Count: 3,589

For peer review only - http://bmjopen.bmj.com/site/about/guidelines.xhtml

ABSTRACT

Introduction

In the UK, 150,000 people every year experience mid substance Achilles tendinopathy. Typically patients are offered a range of treatment options such as exercise, electrotherapy, injections and surgery. With large variations in current practice, there is a pressing need to establish which treatments are effective and which are not. This is the protocol for a multi-centre randomised trial of platelet rich plasma (PRP) versus placebo injection for patients with Achilles tendinopathy.

Methods and analysis

Adult patients with mid-substance Achilles tendinopathy for longer than three months will be screened. Randomisation will be on a 1:1 basis, stratified by centre and bilateral presentation. Participants will be allocated to either a single PRP injection or placebo injection. A minimum of 240 patients will be recruited into the study; this number will provide 90% power to detect a difference of 12 points in VISA-A score at six months. Quality of life, pain and complications data will be collected at baseline, 2 weeks, 3 months and 6 months post randomisation. The differences between treatment groups will be assessed on an intention-to-treat basis.

Ethics, registration and dissemination

This trial was funded by Versus Arthritis and commenced on 1st September 2015 (Versus Arthritis 20831). National Research Ethic Committee approved this study on 30th October 2015 (15/WM/0359). It was registered on the International Standard Randomised Controlled Trial Number registry with reference number ISRCTN 13254422 on 28th October 2015. The first site opened to recruitment on 27th April 2016. The results of this trial will be submitted to a peer-reviewed journal and will inform clinical practice with regard to the treatment of Achilles tendinopathy.

Keywords

Achilles tendon, tendinopathy, injection, platelet rich plasma

ARTICLE SUMMARY: STRENGHTS AND LIMITATIONS OF THIS STUDY

- Blinding of interventions.
- UK wide trial across a minimum of 20 centres to optimise external validity.

- Primary outcome measure is patient-centred.
- The main limitation is that cost effectiveness analysis was not included.

MAIN TEXT

Introduction

Tendinopathy in the mid-substance of the Achilles tendon occurs because of the failure of the tendon to mediate its repair and degeneration processes [1]. The general population has an incidence of 2.35 per 1000 people, equivalent to approximately 150,000 people in the UK every year [2]. Achilles tendinopathy is characterised by pain and stiffness over the lower portion of the calf, impacting on all weight bearing activities [3].

Patients are offered a range of non-operative treatment options for mid-substance Achilles tendinopathy including exercise, electrotherapy and injections. Of these platelet rich plasma (PRP) injections have gained national and international interest following guidelines published by the UK National Institute for Health and Care Excellence (NICE) in 2009 (updated 2013) [4] and international guidance published by the International Olympic Committee 2010 [5]. Both have highlighted PRP injections as a priority area for research, on the basis that they have the potential to reduce morbidity and the need for surgery. With large variations in current practice, there is a pressing need to establish which non-operative treatments are effective [6-8].

PRP is defined as the plasma fraction of a patient's own blood (autologous blood) having a platelet concentration above baseline. Platelets contribute to healing through the action of growth factors [9]. A Cochrane review of injection therapies for Achilles tendinopathy was published in 2015 [10] and identified only two randomised controlled trials (RCTs) evaluating PRP. The first of these was a feasibility RCT for this study that recruited 20 participants at a single site [3], and the second was an RCT that investigated the incremental benefit of adding PRP injections to eccentric loading exercises for 54 participants at a single site [11]. Following this review two further RCTs were published in 2016 and 2017 [12, 13]. They evaluated 60 and 24 participants and both were single site and compared PRP and eccentric loading to placebo and eccentric loading. Of these four trials only one [12] has documented a significant treatment effect of PRP at 6 months (Mean VISA-A score 19.6 (SD = 4.5) in PRP group and 8.8 (SD =3.3) in placebo group).

For peer review only - http://bmjopen.bmj.com/site/about/guidelines.xhtml

Currently available RCT evidence for mid substance Achilles tendinopathy is limited to studies recruiting at a single site with small sample size (< 60 in each study and only 158 participants in total across all studies), that only evaluate the incremental effect of PRP in addition to pre-specified eccentric exercise based programmes. There is a pressing need for adequately powered, multi-centre trials in this important clinical area.

Preparatory Feasibility Research

Between May 2009 and March 2012, our research group undertook a pilot RCT, funded by the Chartered Society of Physiotherapy, to evaluate the feasibility of a main RCT [3]. This study used a process evaluation model to determine the feasibility and acceptability of trial procedures. This work was completed in consultation with a patient user panel and was presented at a Versus Arthritis Achilles tendon Think Tank in April 2013 to further refine the trial question and procedures.

Research Question

In adults with painful mid-substance Achilles tendinopathy lasting longer than three months, does a single injection of PRP (intra-tendinous injection with a peppering technique) improve VISA-A (Victorian institute of Sport Assessment-Achilles) scores by a minimum of 12 points when compared to a placebo (subcutaneous injection of a dry needle and no peppering technique) injection at six months post injection?

Objectives

Primary Objective

To quantify and draw inferences on observed differences in the VISA-A score between the trial treatment groups at six months post randomisation.

Secondary Objectives

1. To quantify and draw inferences on observed differences in the VISA-A score at three months post randomisation.
2. To identify any differences in health related quality of life measurement (HRQoL) between trial treatment groups at three and six months post randomisation.
3. To identify any differences in pain measurements between trial treatment groups at two weeks, three and six months post randomisation.
4. To report the complication profile of PRP injections at 2 weeks, three and six months post randomisation.

For peer review only - http://bmjopen.bmj.com/site/about/guidelines.xhtml

Methods and analysis

Study design

A single-participant blinded, multi-centre, randomised placebo controlled trial.

Sample size

The primary outcome for the study is the VISA-A score [14]; it has a range between 0 and 100, a lower score indicating more symptoms and greater limitation of physical activity and 100 being asymptomatic. The minimum clinically important difference (MCID) for the VISA-A score lies between 10 and 12 points [11, 12]. From our previous feasibility publication [3], VISA-A scores were observed to be approximately normally distributed with a standard deviation of 26. If the true difference between the experimental and control treatment group means is 12 points, a sample of 100 patients in each group will be required to reject the null hypothesis (population means of the experimental and control groups are equal) with probability 0.9 (90% power). This equates to an effect size of 0.46, which we would consider to be moderate. The Type I error rate (significance level) associated with this test is 5%. Allowing approximately 15% loss to follow-up, this amounts to 240 patients in total.

Outcome measures

Outcome measures will be collected at baseline (pre randomisation) by a suitably qualified member of the research team, face to face at the recruiting site and then at two weeks by telephone direct from Warwick Clinical Trials Unit (WCTU) and three and six months post randomisation by postal questionnaire sent from WCTU or telephone if no postal response is received.

Primary

Currently the VISA-A questionnaire is the only patient reported outcome measure developed with supporting validation and reliability evidence, for this common musculoskeletal presentation [14]. The VISA-A is condition specific and is designed to have greater sensitivity and specificity than general purpose scales in the target population. The VISA-A contains eight questions that cover three domains of pain, function and activity. An asymptomatic person would score 100, the lower the score the greater the disability. It has been shown to have good test-re-test reliability (r=0.93), inter-rater and intra-rater reliability (r=0.90) and construct validity when the mean scores were compered across patient populations with differing ranges of severity, with no evidence of floor or ceiling effects [15]. These data will be collected at baseline three and six months after randomisation, where the six months score is the primary outcome.

For peer review only - http://bmjopen.bmj.com/site/about/guidelines.xhtml

Secondary

Euro-Qol 5 Dimensions Score (EQ-5D-5L [16]) is a generic, validated, cross-disciplinary standardised health utility instrument widely used to assess HRQoL. It has two parts, a visual analogue scale (VAS), which measures self-rated health, and a health status instrument, which will be the focus of this study, consisting of a five-level response (no problems, slight problems, moderate problems, severe problems and extreme problems) for five health domains related to daily activities, (i) mobility, (ii) self-care, (iii) usual activities, (iv) pain and discomfort and (v) anxiety and depression. The five dimensions are combined together to provide a 5-digit number that describes the individual's health state. The 5-digit responses, from the EQ-5D health classifications, are converted into an overall score using a published utility algorithm for the United Kingdom population [17]. It has good test-retest reliability, is simple for participants to use, and gives a single preference-based index value for health status that can be used for broader cost-effectiveness comparative purposes [18]. These data will be collected at baseline, three and six months after randomisation.

Pain is assessed using a visual analogue scale comprising of a horizontal 10cm line in length, anchored by 2 verbal descriptors (No pain and worst imaginable pain) to indicate a participant's pain level on the day on completing the questionnaire [19]. These data will be collected at baseline two weeks, three and six months after randomisation.

Participants will be asked if they have experienced any complications during follow-up data collection at two weeks, three and six months after randomisation. Complications expected and related to the study treatment that are collected at the specified time points consist of bruising and discomfort at the venesection site, syncopal (fainting) episode associated with venesection or tendon injection, infection, mild discomfort and bleeding at the injection site, swelling, skin discolouration and allergic reaction.

Screening and Eligibility

Eligible patients will be identified from foot and ankle clinics by the local principal investigator (PI) and invited to speak to a suitably qualified member of the research team. The research team member will complete the eligibility checklist in conjunction with a suitably qualified member of the clinical team. Screening logs will be collected by the clinical care team in a minimum of 20 National Health Service (NHS) trusts in the UK to assess the main reasons for patient exclusion as well as the number of patients willing to take part.

For peer review only

For peer review only - http://bmjopen.bmj.com/site/about/guidelines.xhtml

Patients will be provided with verbal and written information about the study. Written informed consent will be obtained by a suitably qualified member of the research team, after allowing sufficient time for the patient to consider their decision and ask questions about the trial.

Patients aged 18 years or over with pain at the mid-substance of the Achilles tendon for longer than three months confirmed by ultrasound and/or MRI are eligible for the trial. Patients will be excluded for the following reasons; (i) if there is presence of a systemic conditions (including: diabetes, rheumatoid arthritis, peripheral vascular disease), (ii) if they are unable to adhere to trial procedures, are pregnant or actively trying to become pregnant, or breastfeeding at the time of randomisation, (iii) if they have had prior Achilles tendon surgery or rupture on the index side, have had previous major tendon or ankle injury or deformity to either lower leg, (iv) if they have had a fracture of a long bone in either lower limb in the previous six months, (v) if they have previously been randomised in the study, (vi) if they have previously had PRP treatment into a tendon or have any contraindication to receiving a PRP injection (haemodynamic instability, platelet dysfunction syndrome, active cancer, septicaemia, systemic use of anticoagulant therapy (e.g. warfarin, dabigatran, heparin), local infection at site of the procedure). Low dose Aspirin use (or equivalent) is not a contraindication to receiving a PRP injection, therefore these patients were not excluded from the study.

Patients presenting with bilateral Achilles tendinopathy will be randomised and treated as one unit; i.e. the patient will be randomised rather than the tendon. However an index tendon will be identified (this will be the one the patient perceives to be more severe at the point of randomisation).

Randomisation

Study participants will be randomised strictly sequentially, as they become registered as eligible for randomisation on the telephone system. The treatment group will be allocated by computer using a minimisation algorithm and stratification by centre and laterality (one or both Achilles tendons affected) following a call to a secure, centralised, telephone-based randomisation service. Allocation concealment will be maintained by an independent randomisation team who will be responsible for generation of the sequence and will have no role in the allocation of participants. The randomisation system will allocate each participant a unique trial number.

For peer review only - http://bmjopen.bmj.com/site/about/guidelines.xhtml

Stratification by centre will help ensure that any clustering effect related to the centre itself will be equally distributed in the trial arms. Stratification on the basis of bilateral presentation will also be implemented to account for the poorer outcome associated with this sub population.

Post randomisation withdrawals

Participants may withdraw from the trial treatment and/or the trial at any time without prejudice. Unless a participant explicitly withdraws their consent, they will be followed-up wherever possible and data collected as per the protocol until the end of the trial. For participants explicitly withdrawing consent for follow up procedures, trial data obtained up until the point of withdrawal will be included in the final analysis.

Interventions

Pre-Injection

The PI at each site will identify relevant health care professionals to be trained and will record those who have completed training on the delegation log. Only those individuals listed on the delegation log will be allowed to prepare and administer trial interventions. At each centre the trial training programme will be delivered and documented by the Chief Investigator (Dr Kearney) and WCTU Trial Manager.

Following consent, completion of baseline questionnaires and randomisation, all participants will have approximately 10ml of whole blood withdrawn from the antecubital fossa (vein at the elbow) and 5ml of 2% lignocaine (local anaesthetic) will be injected into the skin overlying the painful tendon area for pain relief; this will be done with the participant in the prone (lying down and facing away) position on a treatment couch.

PRP Injection Procedure

The whole blood will be centrifuged using the study specific Glo PRP system (Glofinn, Salo, Finland). Each centre will be supplied with the same centrifuge system to facilitate standardisation of the intervention.

Although treatment in the prone position means that the participant will be facing away from the health care professional, the syringe will be masked to make sure that the participant cannot see the contents of the syringe. Participants will then have one injection of the prepared platelet layer (approximately 3ml) injected into the Achilles tendon using a standard 'peppering' technique at the

For peer review only

site of the tendon pain. This technique involves a single skin portal and then five penetrations of the tendon.

Placebo Injection Procedure

For the placebo injection, the masked needle will be inserted under the skin, but not into the tendon. The participant will feel the needle but nothing will be injected. There is an active debate pertaining to the treatment effect of needling trauma [10], or the trauma of injecting fluid directly into the tendon. Therefore following discussion with the trial TSC and DMC a decision was made that a true placebo arm would need to avoid these possible treatment effects. The group consensus was therefore not to administer the placebo injection intratendinously and under the skin only.

Post Injection

After both the PRP and placebo treatments all participants will receive the same post injection advice sheet. The post injection advice sheet will inform participants that they may have increased pain for 24-48 hours, after which period they can resume their normal activities as pain allows. It will also include advice on potential adverse events (e.g. infection and reddening of the skin) and what to do if they occur.

Bilateral presentations:

The index tendon will be randomised and managed accordingly. Regarding the non-index tendon the participant will have two options, to have no treatment or to receive a second injection into the non-index tendon.

Technical Failure

In the unlikely event that the project specific centrifuge system fails once blood has been drawn for PRP preparation/Placebo preparation the patient will be allocated to the placebo arm and analysed as a protocol violation.

Quality assurance

Quality assurance checks will be carried out by a member of the trial team to assess compliance with the above intervention preparation and delivery. These checks will be done face to face and remotely using recording devices. Any deviations noted from the outlined trial interventions will be monitored by the oversight committees. If required, further training will be implemented to resolve any inconsistencies.

For peer review only - http://bmjopen.bmj.com/site/about/guidelines.xhtml

Additional quality assurance procedures to verify the quality of the PRP preparation will include research nurses from participating trial sites preparing PRP samples from healthy volunteers. Healthy volunteers will be recruited internally from these trial sites through advertisement in routinely distributed newsletters and posters. All volunteers will receive a screening phone call to be considered and only excluded if they have presence of systemic conditions (including: diabetes, rheumatoid arthritis, peripheral vascular disease); they are pregnant and/or breastfeeding; use of anticoagulant therapy (warfarin, dabigatran, heparin) or are unable to adhere and consent to procedures.

Following consent procedures, healthy volunteers will provide two blood samples of up to 10 ml. Sample one will be kept as a whole blood control sample for analysis and sample two will be processed by the trial research nurses to produce a PRP sample for analysis. Samples will be anonymised and transported to an independent test lab which will conduct cell counts of whole blood and PRP preparations. Red cell, platelet and white cell counts (with full differential count) using a blood counter will be undertaken. All samples will be destroyed after analysis.

Blinding

It will not be possible to blind the research or clinical team involved in treatment preparation or delivery due to the nature of the intervention. All participants will be blinded and not know their treatment allocation through masking of the treatment syringe to prevent them from seeing the contents, as well as by being face-down during the injection. Participants in both groups will wait for approximately 30 minutes (PRP preparation time), and not be in the same room as the centrifuge. If this is not possible the participant will lie in the prone position and the centrifuge will spin without any PRP being prepared.

Adverse event management

Adverse events will be listed on Case Report Form for return to the 'ATM' office. Serious adverse events (SAEs) will be entered onto the SAE reporting form and reported to the central study team. All SAEs that occur between date of randomisation and the two week follow-up point will be followed-up as per protocol until the end of the trial. All SAEs will be reported to the sponsor (University of Warwick), ethics committee and oversight committees.

End of trial

For peer review only

The trial will end when all participants have completed their 6 month follow-up. The trial will be stopped prematurely if mandated by the Ethics Committee, following recommendations from relevant oversight committees or funding for the trial ceases. The Research Ethics Committee will be notified in writing within 90 days when the trial has been concluded or within 15 days if terminated early.

Trial oversight

The Trial Management Group (TMG) will meet monthly and consist of the project staff and co-investigators responsible for the day-to-day running of the trial. The TMG will report to an independent Trial Steering Committee (TSC) who will hold meetings not less than once a year and take responsibility for approval of the protocol, monitoring and supervising the progress of the trial and considering recommendations from the Data Monitoring and Safety Committee (DSMC). The TMG will also report to the independent DSMC, who will hold meetings not less than once a year. Confidential reports containing recruitment, protocol compliance, safety data and interim assessments of outcomes will be reviewed by the DSMC.

Patient and Public Involvement (PPI)

PPI members have been active members on the TMG and TSC, and have reviewed all study materials. At the end of the study they will be contribute to dissemination plans distributed through the trial website and other relevant platforms.

Statistical methods

The primary analysis will compare the VISA-A score at six months follow-up between the two treatment groups on an intention-to-treat (ITT) basis. Mixed effects linear regression will be used with adjustment for design factors (centre and laterality), age, sex and baseline VISA-A score. Centre will be taken as a random effect to allow for possible heterogeneity in patient outcomes. Mean difference with 95% confidence interval (CI) will be reported. Data transformation will be applied to the outcome before the modelling if VISA-A at 6 months is non-normally distributed. A p value <0.05 will be considered statistically significant.

The secondary analysis compares the outcomes including VISA-A at 3 months, EQ-5D-5L visual analogue scale (VAS) and index scores at 3 and 6 months and pain VAS and complication rate at 2 weeks, 3 and 6 months. They will be analysed by ITT using linear regression with and without adjustment for design factors and baseline data. In addition, VISA-A at 6 months will be analysed in

For peer review only - http://bmjopen.bmj.com/site/about/guidelines.xhtml

For peer review only

the following ways: 1) unadjusted linear regression by ITT and 2) adjusted mixed-effects regression by per-protocol to assess the impact of non-compliance. Kaplan Meier plot will be used to compare the time to complication by treatment if sufficient number is observed in each complication class. In case of bilateral tendinopathy (both sides), one randomly select side will be included in the analysis. Although missing data is not expected to be a problem for this study, the missing mechanism will be assessed and imputation may be used when deemed appropriate. The imputed data will be used for a sensitivity analysis. Pre-specified subgroup analysis include laterality (single vs bilateral) and duration of symptom (≤median vs >median duration). The analysis will be adjusted in the same way as the primary analysis. A detailed analysis plan will be finalised prior to final data lock.

Ethics and dissemination

National Research Ethic Committee approved this study on 30th October 2015 (15/WM/0359). It was registered on the International Standard Randomised Controlled Trial Number registry with reference number ISRCTN 13254422 on 28th October 2015. The first site opened to recruitment 27th April 2016. PRP injections, amongst others, are currently used in clinical practice for mid substance Achilles tendinopathy and do not expose trial participants to any substantial risks over and above standard care currently received.

The trial will be conducted in full conformance with the principles of the Declaration of Helsinki and to Good Clinical Practice guidelines and comply with all applicable UK legislation and Warwick University Standard Operating Procedures (SOPs). All data will be stored securely and held in accordance with applicable data protection legislation.The trial will be reported in line with the CONSORT statement and Minimum Information for Studies Evaluating Biologics in Orthopaedics (MIBO): Platelet-Rich Plasma and Mesenchymal Stem Cells [20, 21].

The results of this project will be disseminated through peer-reviewed journals, conference presentations amongst the orthopaedic and rehabilitation networks, policy makers such as NICE, patient specific newsletters and through local mechanisms at all participating centres.

For peer review only - http://bmjopen.bmj.com/site/about/guidelines.xhtml

Author contribution and Acknowledgements

RSK , MLC, NP, JY wrote the background section and developed the research question. RSK , MLC, NP, JB wrote the research methodology and management sections of the protocol. NP, CJ and JW wrote the sample size and statistical analysis sections of the protocol. All authors reviewed and approved the final manuscript.

The research team would like to acknowledge Karen Keates for her contribution throughout the feasibility and main trial as a PPI Representative.

Funding

This trial was funded by Versus Arthritis commencing 1st September 2015 (Versus Arthritis 20831).

Disclaimer

The funder has not been involved in the design of the study. The views expressed are those of the authors and not necessarily those of the NHS or the funder.

Competing interests

None

Data Statement

The data sharing plans for the current study will be updated via ISRCTN

Provenance and peer review

Not commissioned; peer reviewed for ethical and funding approval prior to submission.

References

1. Riley, G., *Tendinopathy-from basic science to treatment.* Nature Clinical Practice Rheumatology, 2008. **4**(2): p. 82-89.
2. de Jonge, S., et al., *Incidence of midportion Achilles tendinopathy in the general population.* Br J Sports Med, 2011. **45**(13): p. 1026-8.

For peer review only

3. Kearney, R.S., N. Parsons, and M.L. Costa, *Achilles tendinopathy management: A pilot randomised controlled trial comparing platelet-richplasma injection with an eccentric loading programme.* Bone Joint Res, 2013. **2**(10): p. 227-32.

4. Excellence, N.I.f.H.a.C., *Autologous blood injection for tendinopathy.* National Institute for Health and Care Excellence, 2013.

5. Engebretsen, L., et al., *IOC consensus paper on the use of platelet-rich plasma in sports medicine.* Br J Sports Med, 2010. **44**(15): p. 1072-81.

6. Andres, B.M. and G.A. Murrell, *Treatment of tendinopathy: what works, what does not, and what is on the horizon.* Clin Orthop Relat Res, 2008. **466**(7): p. 1539-54.

7. Sussmilch-Leitch, S.P., et al., *Physical therapies for Achilles tendinopathy: systematic review and meta-analysis.* J Foot Ankle Res, 2012. **5**(1): p. 15.

8. Magnussen, R.A., W.R. Dunn, and A.B. Thomson, *Nonoperative treatment of midportion Achilles tendinopathy: a systematic review.* Clin J Sport Med, 2009. **19**(1): p. 54-64.

9. Alsousou, J., et al., *Platelet rich Plasma in Achilles Tendon Healing 2 (PATH-2) trial: protocol for a multicentre, participant and assessor-blinded, parallel-group randomised clinical trial comparing platelet-rich plasma (PRP) injection versus placebo injection for Achilles tendon rupture.* BMJ Open, 2017. **7**(11): p. e018135.

10. Kearney, R.S., et al., *Injection therapies for Achilles tendinopathy.* Cochrane Database Syst Rev, 2015. **5**: p. CD010960.

11. de Vos, R.J., et al., *Platelet-rich plasma injection for chronic Achilles tendinopathy: a randomized controlled trial.* JAMA, 2010. **303**(2): p. 144-9.

12. Boesen, A.P., et al., *Effect of High-Volume Injection, Platelet-Rich Plasma, and Sham Treatment in Chronic Midportion Achilles Tendinopathy: A Randomized Double-Blinded Prospective Study.* Am J Sports Med, 2017. **45**(9): p. 2034-2043.

For peer review only

For peer review only - http://bmjopen.bmj.com/site/about/guidelines.xhtml

13. Krogh, T.P., et al., *Ultrasound-Guided Injection Therapy of Achilles Tendinopathy With Platelet-Rich Plasma or Saline: A Randomized, Blinded, Placebo-Controlled Trial.* Am J Sports Med, 2016. **44**(8): p. 1990-7.

14. Robinson, J.M., et al., *The VISA-A questionnaire: a valid and reliable index of the clinical severity of Achilles tendinopathy.* Br J Sports Med, 2001. **35**(5): p. 335-41.

15. Iversen, J.V., E.M. Bartels, and H. Langberg, *The victorian institute of sports assessment - achilles questionnaire (visa-a) - a reliable tool for measuring achilles tendinopathy.* Int J Sports Phys Ther, 2012. **7**(1): p. 76-84.

16. Brooks, R., *EuroQol: the current state of play.* Health Policy, 1996. **37**(1): p. 53-72.

17. Dolan, P., *Modeling valuations for EuroQol health states.* Med Care, 1997. **35**(11): p. 1095-108.

18. Herdman, M., et al., *Development and preliminary testing of the new five-level version of EQ-5D (EQ-5D-5L).* Qual Life Res, 2011. **20**(10): p. 1727-36.

19. Hawker, G.A., et al., *Measures of adult pain: Visual Analog Scale for Pain (VAS Pain), Numeric Rating Scale for Pain (NRS Pain), McGill Pain Questionnaire (MPQ), Short-Form McGill Pain Questionnaire (SF-MPQ), Chronic Pain Grade Scale (CPGS), Short Form-36 Bodily Pain Scale (SF-36 BPS), and Measure of Intermittent and Constant Osteoarthritis Pain (ICOAP).* Arthritis Care Res (Hoboken), 2011. **63 Suppl 11**: p. S240-52.

20. Schulz, K.F., et al., *CONSORT 2010 statement: updated guidelines for reporting parallel group randomised trials.* BMJ, 2010. **340**: p. c332.

21. Murray, I.R., et al., *Minimum Information for Studies Evaluating Biologics in Orthopaedics (MIBO): Platelet-Rich Plasma and Mesenchymal Stem Cells.* J Bone Joint Surg Am, 2017. **99**(10): p. 809-819.

For peer review only

For peer review only - http://bmjopen.bmj.com/site/about/guidelines.xhtml

Reporting checklist for protocol of a clinical trial.

Based on the SPIRIT guidelines.

Instructions to authors

Complete this checklist by entering the page numbers from your manuscript where readers will find each of the items listed below.

Your article may not currently address all the items on the checklist. Please modify your text to include the missing information. If you are certain that an item does not apply, please write "n/a" and provide a short explanation.

Upload your completed checklist as an extra file when you submit to a journal.

In your methods section, say that you used the SPIRITreporting guidelines, and cite them as:

Chan A-W, Tetzlaff JM, Altman DG, Laupacis A, Gøtzsche PC, Krleža-Jerić K, Hróbjartsson A, Mann H, Dickersin K, Berlin J, Doré C, Parulekar W, Summerskill W, Groves T, Schulz K, Sox H, Rockhold FW, Rennie D, Moher D. SPIRIT 2013 Statement: Defining standard protocol items for clinical trials. Ann Intern Med. 2013;158(3):200-207

		Reporting Item	Page Number
**Administrative information**			
Title	#1	Descriptive title identifying the study design, population, interventions, and, if applicable, trial acronym	1

For peer review only

Trial registration	#2a	Trial identifier and registry name. If not yet registered, name of intended registry	2 & 12
Trial registration: data set	#2b	All items from the World Health Organization Trial Registration Data Set	2 &12
Protocol version	#3	Date and version identifier	2
Funding	#4	Sources and types of financial, material, and other support	2 and 13
Roles and responsibilities: contributorship	#5a	Names, affiliations, and roles of protocol contributors	13
Roles and responsibilities: sponsor contact information	#5b	Name and contact information for the trial sponsor	10
Roles and responsibilities: sponsor and funder	#5c	Role of study sponsor and funders, if any, in study design; collection, management, analysis, and interpretation of data; writing of the report; and the decision to submit the report for publication, including whether they will have ultimate authority over any of these activities	10 &13
Roles and responsibilities: committees	#5d	Composition, roles, and responsibilities of the coordinating centre, steering committee, endpoint adjudication committee, data management team, and	11

For peer review only

		other individuals or groups overseeing the trial, if applicable (see Item 21a for data monitoring committee)	
**Introduction**			
Background and rationale	#6a	Description of research question and justification for undertaking the trial, including summary of relevant studies (published and unpublished) examining benefits and harms for each intervention	3 & 4
Background and rationale: choice of comparators	#6b	Explanation for choice of comparators	8 & 9
Objectives	#7	Specific objectives or hypotheses	4
Trial design	#8	Description of trial design including type of trial (eg, parallel group, crossover, factorial, single group), allocation ratio, and framework (eg, superiority, equivalence, non-inferiority, exploratory)	5
**Methods: Participants, interventions, and outcomes**			
Study setting	#9	Description of study settings (eg, community clinic, academic hospital) and list of countries where data will be collected. Reference to where list of study sites can be obtained	6

For peer review only

Eligibility criteria	#10	Inclusion and exclusion criteria for participants. If applicable, eligibility criteria for study centres and individuals who will perform the interventions (eg, surgeons, psychotherapists)	6 & 7
Interventions: description	#11a	Interventions for each group with sufficient detail to allow replication, including how and when they will be administered	8 & 9
Interventions: modifications	#11b	Criteria for discontinuing or modifying allocated interventions for a given trial participant (eg, drug dose change in response to harms, participant request, or improving / worsening disease)	9
Interventions: adherance	#11c	Strategies to improve adherence to intervention protocols, and any procedures for monitoring adherence (eg, drug tablet return; laboratory tests)	9 & 10
Interventions: concomitant care	#11d	Relevant concomitant care and interventions that are permitted or prohibited during the trial	7
Outcomes	#12	Primary, secondary, and other outcomes, including the specific measurement variable (eg, systolic blood pressure), analysis metric (eg, change from baseline, final value, time to event), method of aggregation (eg, median, proportion), and time point for each outcome. Explanation of the clinical relevance of chosen efficacy and harm outcomes is strongly recommended	5 & 6

For peer review only

Participant timeline	#13	Time schedule of enrolment, interventions (including any run-ins and washouts), assessments, and visits for participants. A schematic diagram is highly recommended (see Figure)	5 & 6
Sample size	#14	Estimated number of participants needed to achieve study objectives and how it was determined, including clinical and statistical assumptions supporting any sample size calculations	5
Recruitment	#15	Strategies for achieving adequate participant enrolment to reach target sample size	6
**Methods: Assignment of interventions (for controlled trials)**			
Allocation: sequence generation	#16a	Method of generating the allocation sequence (eg, computer-generated random numbers), and list of any factors for stratification. To reduce predictability of a random sequence, details of any planned restriction (eg, blocking) should be provided in a separate document that is unavailable to those who enrol participants or assign interventions	7
Allocation concealment mechanism	#16b	Mechanism of implementing the allocation sequence (eg, central telephone; sequentially numbered, opaque,	7

For peer review only

		sealed envelopes), describing any steps to conceal the sequence until interventions are assigned	
Allocation: implementation	#16c	Who will generate the allocation sequence, who will enrol participants, and who will assign participants to interventions	7
Blinding (masking)	#17a	Who will be blinded after assignment to interventions (eg, trial participants, care providers, outcome assessors, data analysts), and how	10
Blinding (masking): emergency unblinding	#17b	If blinded, circumstances under which unblinding is permissible, and procedure for revealing a participant's allocated intervention during the trial	10
**Methods: Data collection, management, and analysis**			
Data collection plan	#18a	Plans for assessment and collection of outcome, baseline, and other trial data, including any related processes to promote data quality (eg, duplicate measurements, training of assessors) and a description of study instruments (eg, questionnaires, laboratory tests) along with their reliability and validity, if known. Reference to where data collection forms can be found, if not in the protocol	5 & 6

For peer review only

Data collection plan: retention	#18b	Plans to promote participant retention and complete follow-up, including list of any outcome data to be collected for participants who discontinue or deviate from intervention protocols	5 & 6
Data management	#19	Plans for data entry, coding, security, and storage, including any related processes to promote data quality (eg, double data entry; range checks for data values). Reference to where details of data management procedures can be found, if not in the protocol	5 & 12
Statistics: outcomes	#20a	Statistical methods for analysing primary and secondary outcomes. Reference to where other details of the statistical analysis plan can be found, if not in the protocol	11
Statistics: additional analyses	#20b	Methods for any additional analyses (eg, subgroup and adjusted analyses)	11
Statistics: analysis population and missing data	#20c	Definition of analysis population relating to protocol non-adherence (eg, as randomised analysis), and any statistical methods to handle missing data (eg, multiple imputation)	11

Methods: Monitoring

Data monitoring: formal committee	#21a	Composition of data monitoring committee (DMC); summary of its role and reporting structure; statement of whether it is independent from the sponsor and competing interests; and reference to where further	11

For peer review only

		details about its charter can be found, if not in the protocol. Alternatively, an explanation of why a DMC is not needed	
Data monitoring: interim analysis	#21b	Description of any interim analyses and stopping guidelines, including who will have access to these interim results and make the final decision to terminate the trial	N/A
Harms	#22	Plans for collecting, assessing, reporting, and managing solicited and spontaneously reported adverse events and other unintended effects of trial interventions or trial conduct	10
Auditing	#23	Frequency and procedures for auditing trial conduct, if any, and whether the process will be independent from investigators and the sponsor	12
**Ethics and dissemination**			
Research ethics approval	#24	Plans for seeking research ethics committee / institutional review board (REC / IRB) approval	12
Protocol amendments	#25	Plans for communicating important protocol modifications (eg, changes to eligibility criteria, outcomes, analyses) to relevant parties (eg, investigators, REC / IRBs, trial participants, trial registries, journals, regulators)	12

For peer review only

Consent or assent	#26a	Who will obtain informed consent or assent from potential trial participants or authorised surrogates, and how (see Item 32)	6
6Consent or assent: ancillary studies	#26b	Additional consent provisions for collection and use of participant data and biological specimens in ancillary studies, if applicable	9 & 10
Confidentiality	#27	How personal information about potential and enrolled participants will be collected, shared, and maintained in order to protect confidentiality before, during, and after the trial	12
Declaration of interests	#28	Financial and other competing interests for principal investigators for the overall trial and each study site	13
Data access	#29	Statement of who will have access to the final trial dataset, and disclosure of contractual agreements that limit such access for investigators	13
Ancillary and post trial care	#30	Provisions, if any, for ancillary and post-trial care, and for compensation to those who suffer harm from trial participation	12
Dissemination policy: trial results	#31a	Plans for investigators and sponsor to communicate trial results to participants, healthcare professionals, the public, and other relevant groups (eg, via publication, reporting in results databases, or other data sharing arrangements), including any publication restrictions	12

For peer review only

Dissemination policy: authorship	#31b	Authorship eligibility guidelines and any intended use of professional writers	12
Dissemination policy: reproducible research	#31c	Plans, if any, for granting public access to the full protocol, participant-level dataset, and statistical code	12

Appendices

Informed consent materials	#32	Model consent form and other related documentation given to participants and authorised surrogates	8 & 9
Biological specimens	#33	Plans for collection, laboratory evaluation, and storage of biological specimens for genetic or molecular analysis in the current trial and for future use in ancillary studies, if applicable	9 & 10

None The SPIRIT checklist is distributed under the terms of the Creative Commons Attribution License CC-BY-ND 3.0. This checklist can be completed online using https://www.goodreports.org/, a tool made by the EQUATOR Network in collaboration with Penelope.ai

For peer review only

BMJ Open

Platelet Rich Plasma versus Placebo for the management of Achilles Tendinopathy: Protocol for the UK study of Achilles Tendinopathy Management (ATM) multi-centre randomised trial

Journal:	*BMJ Open*
Manuscript ID	bmjopen-2019-034076.R1
Article Type:	Protocol
Date Submitted by the Author:	01-Nov-2019

Complete List of Authors:	Kearney, Rebecca; University of Warwick, Warwick Clinical Trials Unit<br>Parsons, Nicholas; University of Warwick, Warwick Medical School<br>Ji, Chen; University of Warwick, Warwick Clinical Trials Unit<br>Warwick, Jane; University of Warwick, Warwick Clinical Trials Unit<br>Brown, Jaclyn; University of Warwick, Warwick Clinical Trials Unit<br>Young, Jonathan; University Hospitals Coventry and Warwickshire NHS Trust<br>Costa, Matthew; University of Oxford, Nuffield Department of Orthopaedics, Rheumatology, and Musculoskeletal Sciences

**Primary Subject Heading**:	Rehabilitation medicine
Secondary Subject Heading:	Evidence based practice, Research methods
Keywords:	Achilles tendon, Tendinopathy, Injection, Platlet Rich Plasma

SCHOLARONE™
Manuscripts

TITLE PAGE

Title

Platelet Rich Plasma versus Placebo for the management of Achilles Tendinopathy: Protocol for the UK study of Achilles Tendinopathy Management (ATM) multi-centre randomised trial

Corresponding Author

Rebecca S Kearney: Associate Professor, University of Warwick, Clinical Trials Unit, Gibbet Hill Road, Coventry, CV4 7AL; R.S.Kearney@warwick.ac.uk; 02476 573156

Authors

- Nicholas Parsons: Associate Professor, University of Warwick, Warwick Medical School, Gibbet Hill Road, Coventry, CV4 7AL (Nick.Parsons@warwick.ac.uk)
- Chen Ji: Senior Research Fellow, University of Warwick, Clinical Trials Unit, Gibbet Hill Road, Coventry, CV4 7AL (C.J.3@warwick.ac.uk)
- Jane Warwick: Professor, University of Warwick, Clinical Trials Unit, Gibbet Hill Road, Coventry, CV4 7AL (J.Warwick@warwick.ac.uk)
- Jaclyn Brown: Senior Project Manager, University of Warwick, Clinical Trials Unit, Gibbet Hill Road, Coventry, CV4 7AL (J.Brown.10@warwick.ac.uk)
- Jonathan Young: Consultant Orthopaedic Surgeon, University Hospitals Coventry & Warwickshire NHS Trust, Trauma and Orthopaedics, Clifford Bridge Road, Coventry, CV2 2DX (Email: jonathan.young3@uhcw.nhs.uk)
- Matthew Costa: Professor Orthopaedic Trauma, Trauma Unit, Kadoorie Centre, John Radcliffe Hospital, Oxford, OX3 9DU, UK (matthew.costa@ndorms.ox.ac.uk)

Word Count: 3,748

For peer review only - http://bmjopen.bmj.com/site/about/guidelines.xhtml

ABSTRACT

Introduction

In the UK, 150,000 people every year experience mid substance Achilles tendinopathy. Typically patients are offered a range of treatment options such as exercise, electrotherapy, injections and surgery. With large variations in current practice, there is a pressing need to establish which treatments are effective and which are not. This is the protocol for a multi-centre randomised trial of platelet rich plasma (PRP) versus placebo injection for patients with Achilles tendinopathy.

Methods and analysis

Adult patients with mid-substance Achilles tendinopathy for longer than three months will be screened. Randomisation will be on a 1:1 basis, stratified by centre and bilateral presentation. Participants will be allocated to either a single PRP injection or placebo injection. A minimum of 240 patients will be recruited into the study; this number will provide 90% power to detect a difference of 12 points in VISA-A score at six months. Quality of life, pain and complications data will be collected at baseline, 2 weeks, 3 months and 6 months post randomisation. The differences between treatment groups will be assessed on an intention-to-treat basis.

Ethics, registration and dissemination

This trial was funded by Versus Arthritis and commenced on 1st September 2015 (Versus Arthritis 20831). National Research Ethic Committee approved this study on 30th October 2015 (15/WM/0359). It was registered on the International Standard Randomised Controlled Trial Number registry with reference number ISRCTN 13254422 on 28th October 2015. The first site opened to recruitment on 27th April 2016 and the trial was in active recruitment at the point of submitting the protocol paper. The results of this trial will be submitted to a peer-reviewed journal and will inform clinical practice with regard to the treatment of Achilles tendinopathy.

Keywords

Achilles tendon, tendinopathy, injection, platelet rich plasma

ARTICLE SUMMARY: STRENGHTS AND LIMITATIONS OF THIS STUDY

- Blinding of interventions.
- UK wide trial across a minimum of 20 centres to optimise external validity.

- Primary outcome measure is patient-centred.
- The main limitation is that cost effectiveness analysis was not included.

MAIN TEXT

Introduction

Tendinopathy in the mid-substance of the Achilles tendon occurs because of the failure of the tendon to mediate its repair and degeneration processes [1]. The general population has an incidence of 2.35 per 1000 people, equivalent to approximately 150,000 people in the UK every year [2]. Achilles tendinopathy is characterised by pain and stiffness over the lower portion of the calf (mid-portion of the Achilles) impacting on all weight bearing activities [3].

Patients are offered a range of non-operative treatment options for mid-substance Achilles tendinopathy including exercise, electrotherapy and injections. Of these platelet rich plasma (PRP) injections have gained national and international interest following guidelines published by the UK National Institute for Health and Care Excellence (NICE) in 2009 (updated 2013) [4] and international guidance published by the International Olympic Committee 2010 [5]. Both have highlighted PRP injections as a priority area for research, on the basis that they have the potential to reduce morbidity and the need for surgery. With large variations in current practice, there is a pressing need to establish which non-operative treatments are effective [6-8].

PRP is defined as the plasma fraction of a patient's own blood (autologous blood) having a platelet concentration above baseline. Platelets contribute to healing through the action of growth factors [9]. A Cochrane review of injection therapies for Achilles tendinopathy was published in 2015 [10] and identified only two randomised controlled trials (RCTs) evaluating PRP. The first of these was a feasibility RCT for this study that recruited 20 participants at a single site [3], and the second was an RCT that investigated the incremental benefit of adding PRP injections to eccentric loading exercises for 54 participants at a single site [11]. Following this review two further RCTs were published in 2016 and 2017 [12, 13]. They evaluated 60 and 24 participants and both were single site and compared PRP and eccentric loading to placebo and eccentric loading. Of these four trials only one [12] has documented a significant treatment effect of PRP at 6 months (Mean VISA-A score 19.6 (SD = 4.5) in PRP group and 8.8 (SD =3.3) in placebo group).

For peer review only - http://bmjopen.bmj.com/site/about/guidelines.xhtml

Currently available RCT evidence for mid substance Achilles tendinopathy is limited to studies recruiting at a single site with small sample size (< 60 in each study and only 158 participants in total across all studies), that only evaluate the incremental effect of PRP in addition to pre-specified eccentric exercise based programmes. No studies have evaluated the effectiveness of PRP across multiple sites, in an adequately powered trial. There is a pressing need for adequately powered, multi-centre trials in this important clinical area. Which this project will address.

Preparatory Feasibility Research

Between May 2009 and March 2012, our research group undertook a pilot RCT, funded by the Chartered Society of Physiotherapy, to evaluate the feasibility of a main RCT [3]. This study used a process evaluation model to determine the feasibility and acceptability of trial procedures. This work was completed in consultation with a patient user panel and was presented at a Versus Arthritis Achilles tendon Think Tank in April 2013 to further refine the trial question and procedures.

Research Question

In adults with painful mid-substance Achilles tendinopathy lasting longer than three months, does a single injection of PRP (intra-tendinous injection with a peppering technique) improve VISA-A (Victorian institute of Sport Assessment-Achilles) scores by a minimum of 12 points when compared to a placebo (subcutaneous injection of a dry needle and no peppering technique) injection at six months post injection?

Objectives

Primary Objective

To quantify and draw inferences on observed differences in the VISA-A score between the trial treatment groups at six months post randomisation.

Secondary Objectives

1. To quantify and draw inferences on observed differences in the VISA-A score at three months post randomisation.
2. To identify any differences in health related quality of life measurement (HRQoL) between trial treatment groups at three and six months post randomisation.
3. To identify any differences in pain measurements between trial treatment groups at two weeks, three and six months post randomisation.

For peer review only - http://bmjopen.bmj.com/site/about/guidelines.xhtml

4. To report the complication profile of PRP injections at 2 weeks, three and six months post randomisation.

Methods and analysis

Study design

A single-participant blinded, multi-centre, randomised placebo controlled trial. Ethical approvals have been obtained from all participating sites (Supplementary file 1).

Sample size

The primary outcome for the study is the VISA-A score [14]; it has a range between 0 and 100, a lower score indicating more symptoms and greater limitation of physical activity and 100 being asymptomatic. The minimum clinically important difference (MCID) for the VISA-A score is estimated to lie between 10 and 12 points [11, 12]. From our previous feasibility publication [3], VISA-A scores were observed to be approximately normally distributed with a standard deviation of 26. If the true difference between the experimental and control treatment group means is 12 points, a sample of 100 patients in each group will be required to reject the null hypothesis (population means of the experimental and control groups are equal) with probability 0.9 (90% power). This equates to an effect size of 0.46, which we would consider to be moderate. The Type I error rate (significance level) associated with this test is 5%. Allowing approximately 15% loss to follow-up, this amounts to 240 patients in total.

Outcome measures

Outcome measures will be collected at baseline (pre randomisation) by a suitably qualified member of the research team, face to face at the recruiting site. Baseline participant characteristics to be collected include duration of symptoms, side affected (left, right, bilateral), previous treatments for the Achilles tendinopathy, current medications, weight, height, age, gender, ethnicity, smoking and drinking status and employment status. Participants will then be followed up at two weeks by telephone direct from Warwick Clinical Trials Unit (WCTU) and three and six months post randomisation by postal questionnaire sent from WCTU or telephone if no postal response is received (Supplementary file 2).

Primary

Currently the VISA-A questionnaire is the only patient reported outcome measure developed with supporting validation and reliability evidence, for this common musculoskeletal presentation [14].

For peer review only

For peer review only - http://bmjopen.bmj.com/site/about/guidelines.xhtml

The VISA-A is condition specific and is designed to have greater sensitivity and specificity than general purpose scales in the target population. The VISA-A contains eight questions that cover three domains of pain, function and activity. An asymptomatic person would score 100, the lower the score the greater the disability. It has been shown to have good test-re-test reliability (r=0.93), inter-rater and intra-rater reliability (r=0.90) and construct validity when the mean scores were compared across patient populations with differing ranges of severity, with no evidence of floor or ceiling effects [15]. These data will be collected at baseline three and six months after randomisation, where the six months score is the primary outcome.

Secondary

Euro-Qol 5 Dimensions Score (EQ-5D-5L [16]) is a generic, validated, cross-disciplinary standardised health utility instrument widely used to assess HRQoL. It has two parts, a visual analogue scale (VAS), which measures self-rated health, and a health status instrument, which will be the focus of this study, consisting of a five-level response (no problems, slight problems, moderate problems, severe problems and extreme problems) for five health domains related to daily activities, (i) mobility, (ii) self-care, (iii) usual activities, (iv) pain and discomfort and (v) anxiety and depression. The five dimensions are combined together to provide a 5-digit number that describes the individual's health state. The 5-digit responses, from the EQ-5D health classifications, are converted into an overall score using a published utility algorithm for the United Kingdom population [17]. It has good test-retest reliability, is simple for participants to use, and gives a single preference-based index value for health status that can be used for broader cost-effectiveness comparative purposes [18]. These data will be collected at baseline, three and six months after randomisation.

Pain is assessed using a visual analogue scale comprising of a horizontal 10cm line in length, anchored by 2 verbal descriptors (No pain and worst imaginable pain) to indicate a participant's pain level on the day on completing the questionnaire [19]. These data will be collected at baseline two weeks, three and six months after randomisation.

Participants will be asked if they have experienced any complications during follow-up data collection at two weeks, three and six months after randomisation. Complications expected and related to the study treatment that are collected at the specified time points consist of bruising and discomfort at the venesection site, syncopal (fainting) episode associated with venesection or tendon injection, infection, mild discomfort and bleeding at the injection site, swelling, skin discolouration and allergic reaction.

For peer review only - http://bmjopen.bmj.com/site/about/guidelines.xhtml

For peer review only

Screening and Eligibility

Eligible patients will be identified from foot and ankle clinics by the local principal investigator (PI) and invited to speak to a suitably qualified member of the research team. The research team member will complete the eligibility checklist in conjunction with a suitably qualified member of the clinical team. Screening logs will be collected by the clinical care team in a minimum of 20 National Health Service (NHS) trusts in the UK to assess the main reasons for patient exclusion as well as the number of patients willing to take part.

Patients will be provided with verbal and written information about the study. Written informed consent will be obtained (Supplementary file 3) by a suitably qualified member of the research team, after allowing sufficient time for the patient to consider their decision and ask questions about the trial.

Patients aged 18 years or over with pain at the mid-substance of the Achilles tendon for longer than three months confirmed by ultrasound and/or MRI are eligible for the trial. Patients will be excluded for the following reasons; (i) if there is presence of a systemic conditions (including: diabetes, rheumatoid arthritis, peripheral vascular disease), (ii) if they are unable to adhere to trial procedures, are pregnant or actively trying to become pregnant, or breastfeeding at the time of randomisation, (iii) if they have had prior Achilles tendon surgery or rupture on the index side, have had previous major tendon or ankle injury or deformity to either lower leg, (iv) if they have had a fracture of a long bone in either lower limb in the previous six months, (v) if they have previously been randomised in the study, (vi) if they have previously had PRP treatment into a tendon or have any contraindication to receiving a PRP injection (haemodynamic instability, platelet dysfunction syndrome, active cancer, septicaemia, systemic use of anticoagulant therapy (e.g. warfarin, dabigatran, heparin), local infection at site of the procedure). Low dose Aspirin use (or equivalent) is not a contraindication to receiving a PRP injection, therefore these patients will not be excluded from the study.

Patients presenting with bilateral Achilles tendinopathy will be randomised and treated as one unit; i.e. the patient will be randomised rather than the tendon. However an index tendon will be identified (this will be the one the patient perceives to be more severe at the point of randomisation).

Randomisation

For peer review only

Study participants will be randomised strictly sequentially, as they become registered as eligible for randomisation on the telephone system. The treatment group will be allocated by computer using a minimisation algorithm and stratification by centre and laterality (one or both Achilles tendons affected) following a call to a secure, centralised, telephone-based randomisation service. Allocation concealment will be maintained by an independent randomisation team who will be responsible for generation of the sequence and will have no role in the allocation of participants. The randomisation system will allocate each participant a unique trial number.

Stratification by centre will help ensure that any clustering effect related to the centre itself will be equally distributed in the trial arms. Stratification on the basis of bilateral presentation will also be implemented to account for the poorer outcome associated with this sub population.

Post randomisation withdrawals

Participants may withdraw from the trial treatment and/or the trial at any time without prejudice. Unless a participant explicitly withdraws their consent, they will be followed-up wherever possible and data collected as per the protocol until the end of the trial. For participants explicitly withdrawing consent for follow up procedures, trial data obtained up until the point of withdrawal will be included in the final analysis.

Interventions

Pre-Injection

The PI at each site will identify relevant health care professionals to be trained and will record those who have completed training on the delegation log. Only those individuals listed on the delegation log will be allowed to prepare and administer trial interventions. At each centre the trial training programme will be delivered and documented by the Chief Investigator (Dr Kearney) and WCTU Trial Manager.

Following consent, completion of baseline questionnaires and randomisation, all participants will have approximately 10ml of whole blood withdrawn from the antecubital fossa (vein at the elbow) and 5ml of 2% lignocaine (local anaesthetic) will be injected into the skin overlying the painful tendon area for pain relief; this will be done with the participant in the prone (lying down and facing away) position on a treatment couch.

PRP Injection Procedure

For peer review only - http://bmjopen.bmj.com/site/about/guidelines.xhtml

The whole blood will be centrifuged using the study specific Glo PRP system (Glofinn, Salo, Finland). Each centre will be supplied with the same centrifuge system to facilitate standardisation of the intervention.

Although treatment in the prone position means that the participant will be facing away from the health care professional, the syringe will be masked to make sure that the participant cannot see the contents of the syringe. Participants will then have one injection of the prepared platelet layer (approximately 3ml) injected into the Achilles tendon using a standard 'peppering' technique at the site of the tendon pain. This technique involves a single skin portal and then five penetrations of the tendon.

Placebo Injection Procedure

For the placebo injection, the masked needle will be inserted under the skin, but not into the tendon. The participant will feel the needle but nothing will be injected. There is an active debate pertaining to the treatment effect of needling trauma [10], or the trauma of injecting fluid directly into the tendon. Therefore following discussion with the Trial Steering Committee (TSC) and the Data Monitoring and Safety Committee (DSMC) a decision was made that a true placebo arm would need to avoid these possible treatment effects. The group consensus was therefore not to administer the placebo injection intratendinously and under the skin only. At the six months time point, following completion of all data collection, participants will be asked if they think they know their treatment allocation or not.

Post Injection

After both the PRP and placebo treatments all participants will receive the same post injection advice sheet. The post injection advice sheet will inform participants that they may have increased pain for 24-48 hours, after which period they can resume their normal activities as pain allows. It will also include advice on potential adverse events (e.g. infection and reddening of the skin) and what to do if they occur. Participants will be instructed not to undergo any further interventions for a period of six months, which will be monitored at each follow up time point.

Bilateral presentations:

The index tendon will be randomised and managed accordingly. Regarding the non-index tendon the participant will have two options, to have no treatment or to receive a second injection into the non-index tendon.

For peer review only

For peer review only - http://bmjopen.bmj.com/site/about/guidelines.xhtml

Technical Failure

In the unlikely event that the project specific centrifuge system fails once blood has been drawn for PRP preparation/Placebo preparation the patient will be allocated to the placebo arm and analysed as a protocol violation.

Quality assurance

Quality assurance checks will be carried out by a member of the trial team to assess compliance with the above intervention preparation and delivery. These checks will be done face to face and remotely using recording devices. Any deviations noted from the outlined trial interventions will be monitored by the oversight committees. If required, further training will be implemented to resolve any inconsistencies.

Additional quality assurance procedures to verify the quality of the PRP preparation will include research nurses from participating trial sites preparing PRP samples from healthy volunteers. Healthy volunteers will be recruited internally from these trial sites through advertisement in routinely distributed newsletters and posters. All volunteers will receive a screening phone call to be considered and only excluded if they have presence of systemic conditions (including: diabetes, rheumatoid arthritis, peripheral vascular disease); they are pregnant and/or breastfeeding; use of anticoagulant therapy (warfarin, dabigatran, heparin) or are unable to adhere and consent to procedures.

Following consent procedures, healthy volunteers will provide two blood samples of up to 10 ml. Sample one will be kept as a whole blood control sample for analysis and sample two will be processed by the trial research nurses to produce a PRP sample for analysis. Samples will be anonymised and transported to an independent test lab which will conduct cell counts of whole blood and PRP preparations. Red cell, platelet and white cell counts (with full differential count) using a blood counter will be undertaken. All samples will be destroyed after analysis.

Blinding

It will not be possible to blind the research or clinical team involved in treatment preparation or delivery due to the nature of the intervention. All participants will be blinded and not know their treatment allocation through masking of the treatment syringe to prevent them from seeing the contents, as well as by being face-down during the injection. Participants in both groups will wait for

For peer review only - http://bmjopen.bmj.com/site/about/guidelines.xhtml

approximately 30 minutes (PRP preparation time), and not be in the same room as the centrifuge. If this is not possible the participant will lie in the prone position and the centrifuge will spin without any PRP being prepared.

Adverse event management

Adverse events will be listed on Case Report Form for return to the 'ATM' office. Serious adverse events (SAEs) will be entered onto the SAE reporting form and reported to the central study team. All SAEs that occur between date of randomisation and the two week follow-up point will be followed-up as per protocol until the end of the trial. All SAEs will be reported to the sponsor (University of Warwick), ethics committee and oversight committees.

End of trial

The trial will end when all participants have completed their 6 month follow-up. The trial will be stopped prematurely if mandated by the Ethics Committee, following recommendations from relevant oversight committees or funding for the trial ceases. The Research Ethics Committee will be notified in writing within 90 days when the trial has been concluded or within 15 days if terminated early.

Trial oversight

The Trial Management Group (TMG) will meet monthly and consist of the project staff and co-investigators responsible for the day-to-day running of the trial. The TMG will report to an independent TSC who will hold meetings not less than once a year and take responsibility for approval of the protocol, monitoring and supervising the progress of the trial and considering recommendations from the DSMC. The TMG will also report to the independent DSMC, who will hold meetings not less than once a year. Confidential reports containing recruitment, protocol compliance, safety data and interim assessments of outcomes will be reviewed by the DSMC.

Patient and Public Involvement (PPI)

PPI members have been active members on the TMG and TSC, and have reviewed all study materials. At the end of the study they will be contribute to dissemination plans distributed through the trial website and other relevant platforms.

Statistical methods

For peer review only - http://bmjopen.bmj.com/site/about/guidelines.xhtml

The primary analysis will compare the VISA-A score at six months follow-up between the two treatment groups on an intention-to-treat (ITT) basis. Mixed effects linear regression will be used with adjustment for design factors (centre and laterality), age, sex and baseline VISA-A score. Centre will be taken as a random effect to allow for possible heterogeneity in patient outcomes. Mean difference with 95% confidence interval (CI) will be reported. Data transformation will be applied to the outcome before the modelling if VISA-A at 6 months is non-normally distributed. A p value <0.05 will be considered statistically significant.

The secondary analysis compares the outcomes including VISA-A at 3 months, EQ-5D-5L visual analogue scale (VAS) and index scores at 3 and 6 months and pain VAS and complication rate at 2 weeks, 3 and 6 months. They will be analysed by ITT using linear regression with and without adjustment for design factors and baseline data. In addition, VISA-A at 6 months will be analysed in the following ways: 1) unadjusted linear regression by ITT and 2) adjusted mixed-effects regression by per-protocol to assess the impact of non-compliance. Kaplan Meier plot will be used to compare the time to complication by treatment if sufficient number is observed in each complication class. In case of bilateral tendinopathy (both sides), one randomly select side will be included in the analysis. Although missing data is not expected to be a problem for this study, the missing mechanism will be assessed and imputation may be used when deemed appropriate. The imputed data will be used for a sensitivity analysis. Pre-specified subgroup analysis include laterality (single vs bilateral) and duration of symptom (≤median vs >median duration). The analysis will be adjusted in the same way as the primary analysis. A detailed analysis plan will be finalised prior to final data lock.

Ethics and dissemination

National Research Ethic Committee approved this study on 30th October 2015 (15/WM/0359). It was registered on the International Standard Randomised Controlled Trial Number registry with reference number ISRCTN 13254422 on 28th October 2015. The first site opened to recruitment 27th April 2016, the trial was in active recruitment at the point of submitting the protocol paper. PRP injections, amongst others, are currently used in clinical practice for mid substance Achilles tendinopathy and do not expose trial participants to any substantial risks over and above standard care currently received.

The trial will be conducted in full conformance with the principles of the Declaration of Helsinki and to Good Clinical Practice guidelines and comply with all applicable UK legislation and Warwick University Standard Operating Procedures (SOPs). All data will be stored securely and held in

For peer review only - http://bmjopen.bmj.com/site/about/guidelines.xhtml

accordance with applicable data protection legislation.The trial will be reported in line with the CONSORT statement and Minimum Information for Studies Evaluating Biologics in Orthopaedics (MIBO): Platelet-Rich Plasma and Mesenchymal Stem Cells [20, 21].

The results of this project will be disseminated through peer-reviewed journals, conference presentations amongst the orthopaedic and rehabilitation networks, policy makers such as NICE, patient specific newsletters and through local mechanisms at all participating centres.

For peer review only

For peer review only - http://bmjopen.bmj.com/site/about/guidelines.xhtml

Author contribution and Acknowledgements

RSK , MLC, NP, JY wrote the background section and developed the research question. RSK , MLC, NP, JB wrote the research methodology and management sections of the protocol. NP, CJ and JW wrote the sample size and statistical analysis sections of the protocol. All authors reviewed and approved the final manuscript.

The research team would like to acknowledge Karen Keates for her contribution throughout the feasibility and main trial as a PPI Representative.

Funding

This trial was funded by Versus Arthritis commencing 1st September 2015 (Versus Arthritis 20831).

Disclaimer

The funder has not been involved in the design of the study. The views expressed are those of the authors and not necessarily those of the NHS or the funder.

Competing interests

None

Data Statement

The data sharing plans for the current study will be updated via ISRCTN

Provenance and peer review

Not commissioned; peer reviewed for ethical and funding approval prior to submission.

References

1. Riley, G., *Tendinopathy-from basic science to treatment.* Nature Clinical Practice Rheumatology, 2008. **4**(2): p. 82-89.
2. de Jonge, S., et al., *Incidence of midportion Achilles tendinopathy in the general population.* Br J Sports Med, 2011. **45**(13): p. 1026-8.

For peer review only

3. Kearney, R.S., N. Parsons, and M.L. Costa, *Achilles tendinopathy management:A pilot randomised controlled trial comparing platelet-richplasma injection with an eccentric loading programme.* Bone Joint Res, 2013. **2**(10): p. 227-32.

4. National Institution for Health and Social Care Excellence (NICE), *Autologous blood injection for tendinopathy.* 2013.

5. Engebretsen, L., et al., *IOC consensus paper on the use of platelet-rich plasma in sports medicine.* Br J Sports Med, 2010. **44**(15): p. 1072-81.

6. Andres, B.M. and G.A. Murrell, *Treatment of tendinopathy: what works, what does not, and what is on the horizon.* Clin Orthop Relat Res, 2008. **466**(7): p. 1539-54.

7. Sussmilch-Leitch, S.P., et al., *Physical therapies for Achilles tendinopathy: systematic review and meta-analysis.* J Foot Ankle Res, 2012. **5**(1): p. 15.

8. Magnussen, R.A., W.R. Dunn, and A.B. Thomson, *Nonoperative treatment of midportion Achilles tendinopathy: a systematic review.* Clin J Sport Med, 2009. **19**(1): p. 54-64.

9. Alsousou, J., et al., *Platelet rich Plasma in Achilles Tendon Healing 2 (PATH-2) trial: protocol for a multicentre, participant and assessor-blinded, parallel-group randomised clinical trial comparing platelet-rich plasma (PRP) injection versus placebo injection for Achilles tendon rupture.* BMJ Open, 2017. **7**(11): p. e018135.

10. Kearney, R.S., et al., *Injection therapies for Achilles tendinopathy.* Cochrane Database Syst Rev, 2015. **5**: p. CD010960.

11. de Vos, R.J., et al., *Platelet-rich plasma injection for chronic Achilles tendinopathy: a randomized controlled trial.* JAMA, 2010. **303**(2): p. 144-9.

12. Boesen, A.P., et al., *Effect of High-Volume Injection, Platelet-Rich Plasma, and Sham Treatment in Chronic Midportion Achilles Tendinopathy: A Randomized Double-Blinded Prospective Study.* Am J Sports Med, 2017. **45**(9): p. 2034-2043.

For peer review only

For peer review only - http://bmjopen.bmj.com/site/about/guidelines.xhtml

13. Krogh, T.P., et al., *Ultrasound-Guided Injection Therapy of Achilles Tendinopathy With Platelet-Rich Plasma or Saline: A Randomized, Blinded, Placebo-Controlled Trial.* Am J Sports Med, 2016. **44**(8): p. 1990-7.

14. Robinson, J.M., et al., *The VISA-A questionnaire: a valid and reliable index of the clinical severity of Achilles tendinopathy.* Br J Sports Med, 2001. **35**(5): p. 335-41.

15. Iversen, J.V., E.M. Bartels, and H. Langberg, *The victorian institute of sports assessment - achilles questionnaire (visa-a) - a reliable tool for measuring achilles tendinopathy.* Int J Sports Phys Ther, 2012. **7**(1): p. 76-84.

16. Brooks, R., *EuroQol: the current state of play.* Health Policy, 1996. **37**(1): p. 53-72.

17. Dolan, P., *Modeling valuations for EuroQol health states.* Med Care, 1997. **35**(11): p. 1095-108.

18. Herdman, M., et al., *Development and preliminary testing of the new five-level version of EQ-5D (EQ-5D-5L).* Qual Life Res, 2011. **20**(10): p. 1727-36.

19. Hawker, G.A., et al., *Measures of adult pain: Visual Analog Scale for Pain (VAS Pain), Numeric Rating Scale for Pain (NRS Pain), McGill Pain Questionnaire (MPQ), Short-Form McGill Pain Questionnaire (SF-MPQ), Chronic Pain Grade Scale (CPGS), Short Form-36 Bodily Pain Scale (SF-36 BPS), and Measure of Intermittent and Constant Osteoarthritis Pain (ICOAP).* Arthritis Care Res (Hoboken), 2011. **63 Suppl 11**: p. S240-52.

20. Schulz, K.F., et al., *CONSORT 2010 statement: updated guidelines for reporting parallel group randomised trials.* BMJ, 2010. **340**: p. c332.

21. Murray, I.R., et al., *Minimum Information for Studies Evaluating Biologics in Orthopaedics (MIBO): Platelet-Rich Plasma and Mesenchymal Stem Cells.* J Bone Joint Surg Am, 2017. **99**(10): p. 809-819.

For peer review only

For peer review only - http://bmjopen.bmj.com/site/about/guidelines.xhtml

List of site codes and open date

Site	Site code	Open Date
University Hospitals Coventry and Warwickshire	UHCW	27/04/2016
The Princess Royal Hospital, Shrewsbury and Telford**	SHTL	10/06/2016
Ninewells Hospital, Dundee	DUND	15/06/2016
Norfolk and Norwich University Hospitals***	NNUH	12/08/2016
University Hospitals Leicester	LEIC	20/07/2016
Northern General Hospital, Sheffield	NGH	26/10/2016
Northumbria Hospital	NORT	04/01/2017
Leighton Hospital*	MCHT	18/01/2017
Morriston Hospital, Swansea	SWAN	04/04/2017
Arrowe Park Hospital	ARRW	28/04/2017
Wexham Park Hospital	WEX	27/06/2017
Royal Liverpool Hospital****	ROYL	17/07/2017
Robert Jones and Agnes Hunt Orthopaedic Hospital	RJAH	22/08/2017
Doncaster and Bassetlaw Hospital	DONC	08/06/2017
Royal Devon and Exeter Hospital	EXET	10/01/2018
Musgrove Park Hospital	MUSG	15/01/2018
Merthyr Tydfil	MERT	13/02/2018
Basildon University Hospital	BASL	05/03/2018
George Eliot Hospital	GEH	02/05/2018
North Tees and Hartlepool Hospital	TEES	19/06/2018
Cardiff and Vale Orthopaedic Centre, Llandough Hospital, Cardiff	CARD	03/07/2018
Alexandra Hospital, Redditch	WAH	26/11/2018
Wharfedale Hospital, Leeds Community Healthcare	LCH	29/11/2018
Imperial College London	IMPC	19/02/2019

*Closed to *recruitment August 2017*

***Closed to recruitment September 2018*

****Closed to recruitment May 2018*

*****Closed to recruitment November 2018*

For peer review only - http://bmjopen.bmj.com/site/about/guidelines.xhtml

For peer review only

Schedule of enrolment, interventions and assessments.

**Visit Window** (No. Weeks ± No. Days)	**Baseline**	**2 wk post randomisation:** telephone follow up	**3 m (± 1 m) after randomisation: postal follow up**	**6 m (± 1m) after randomisation: postal follow up**
Eligibility screen	✓			
Written informed consent	✓			
Baseline data Form	✓			
Randomisation	✓			
Intervention delivery	✓			
Pain score	✓	✓	✓	✓
VISA-A	✓		✓	✓
EQ5D-5L	✓		✓	✓
Adverse events	✓	✓	✓	✓

For peer review only

CONSENT FORM

Achilles Tendinopathy Management (ATM): A multi-centre placebo controlled randomised controlled trial comparing Platelet Rich Plasma (PRP) to placebo (imitation) injection in adults with Achilles tendon pain.

Chief Investigator: Dr. Rebecca Kearney

Site ID | | | | **Participant ID** | | | |

Please INITIAL boxes do not tick

1. I confirm that I have read and understood the information sheet (version 6.1 dated 04-JAN-2019) for the above study. I have had the opportunity to consider the information, ask questions and have had these answered satisfactorily. ☐
2. I understand that my participation is voluntary and that I am free to withdraw at any time, without giving any reason, without my medical care or legal rights being affected. ☐
3. I understand that relevant sections of any of my medical notes and data collected during the study may be looked at by responsible individuals from regulatory authorities or from the NHS Trust, where it is relevant to my taking part in this research. I give permission for these individuals to have access to my records. ☐
4. I understand that appropriate personal identifying information will be collected, stored and used by Warwick Clinical Trials Unit to enable follow up of my health status. This will include the contact details for my next of kin. I understand that any information will be treated with the strictest security and confidentiality. ☐
5. I understand that the information held and maintained by The Health and Social Care Information Centre and other central UK NHS bodies may be used to help contact me or provide information about my health status ☐
6. I agree to have a blood sample taken for the purposes of the above study and that part of this sample will be used in my treatment if I am allocated to the Platelet Rich Plasma (PRP) Injection group. I understand that if I am not allocated to this group, my sample will be destroyed and not used as part of my treatment. ☐
7. I agree that I will complete questionnaires regarding the impact of the treatment on my pain, function and activity and overall quality of life. ☐
8. I agree to my GP being informed of my participation in the study. ☐
9. I agree to being sent text messages to remind me that my questionnaire is due. ☐
10. I agree to take part in the above study. ☐

Full name of patient	Signature	Date (dd/mmm/yyyy)
Full name of person taking consent	Signature	Date (dd/mmm/yyyy)

For peer review only - http://bmjopen.bmj.com/site/about/guidelines.xhtml

Original copy to be kept in in the ATM site file, one copy to be kept in the patients' medical notes, and one copy to the patient.

Reporting checklist for protocol of a clinical trial.

Based on the SPIRIT guidelines.

Instructions to authors

Complete this checklist by entering the page numbers from your manuscript where readers will find each of the items listed below.

Your article may not currently address all the items on the checklist. Please modify your text to include the missing information. If you are certain that an item does not apply, please write "n/a" and provide a short explanation.

Upload your completed checklist as an extra file when you submit to a journal.

In your methods section, say that you used the SPIRITreporting guidelines, and cite them as:

Chan A-W, Tetzlaff JM, Altman DG, Laupacis A, Gøtzsche PC, Krleža-Jerić K, Hróbjartsson A, Mann H, Dickersin K, Berlin J, Doré C, Parulekar W, Summerskill W, Groves T, Schulz K, Sox H, Rockhold FW, Rennie D, Moher D. SPIRIT 2013 Statement: Defining standard protocol items for clinical trials. Ann Intern Med. 2013;158(3):200-207

		Reporting Item	Page Number
**Administrative information**			
Title	#1	Descriptive title identifying the study design, population, interventions, and, if applicable, trial acronym	1

For peer review only

Trial registration	#2a	Trial identifier and registry name. If not yet registered, name of intended registry	2 & 12
Trial registration: data set	#2b	All items from the World Health Organization Trial Registration Data Set	2 &12
Protocol version	#3	Date and version identifier	2
Funding	#4	Sources and types of financial, material, and other support	2 and 13
Roles and responsibilities: contributorship	#5a	Names, affiliations, and roles of protocol contributors	13
Roles and responsibilities: sponsor contact information	#5b	Name and contact information for the trial sponsor	10
Roles and responsibilities: sponsor and funder	#5c	Role of study sponsor and funders, if any, in study design; collection, management, analysis, and interpretation of data; writing of the report; and the decision to submit the report for publication, including whether they will have ultimate authority over any of these activities	10 &13
Roles and responsibilities: committees	#5d	Composition, roles, and responsibilities of the coordinating centre, steering committee, endpoint adjudication committee, data management team, and	11

For peer review only

		other individuals or groups overseeing the trial, if applicable (see Item 21a for data monitoring committee)	
**Introduction**			
Background and rationale	#6a	Description of research question and justification for undertaking the trial, including summary of relevant studies (published and unpublished) examining benefits and harms for each intervention	3 & 4
Background and rationale: choice of comparators	#6b	Explanation for choice of comparators	8 & 9
Objectives	#7	Specific objectives or hypotheses	4
Trial design	#8	Description of trial design including type of trial (eg, parallel group, crossover, factorial, single group), allocation ratio, and framework (eg, superiority, equivalence, non-inferiority, exploratory)	5
**Methods: Participants, interventions, and outcomes**			
Study setting	#9	Description of study settings (eg, community clinic, academic hospital) and list of countries where data will be collected. Reference to where list of study sites can be obtained	6

For peer review only

Eligibility criteria	#10	Inclusion and exclusion criteria for participants. If applicable, eligibility criteria for study centres and individuals who will perform the interventions (eg, surgeons, psychotherapists)	6 & 7
Interventions: description	#11a	Interventions for each group with sufficient detail to allow replication, including how and when they will be administered	8 & 9
Interventions: modifications	#11b	Criteria for discontinuing or modifying allocated interventions for a given trial participant (eg, drug dose change in response to harms, participant request, or improving / worsening disease)	9
Interventions: adherance	#11c	Strategies to improve adherence to intervention protocols, and any procedures for monitoring adherence (eg, drug tablet return; laboratory tests)	9 & 10
Interventions: concomitant care	#11d	Relevant concomitant care and interventions that are permitted or prohibited during the trial	7
Outcomes	#12	Primary, secondary, and other outcomes, including the specific measurement variable (eg, systolic blood pressure), analysis metric (eg, change from baseline, final value, time to event), method of aggregation (eg, median, proportion), and time point for each outcome. Explanation of the clinical relevance of chosen efficacy and harm outcomes is strongly recommended	5 & 6

For peer review only

Participant timeline	#13	Time schedule of enrolment, interventions (including any run-ins and washouts), assessments, and visits for participants. A schematic diagram is highly recommended (see Figure)	5 & 6
Sample size	#14	Estimated number of participants needed to achieve study objectives and how it was determined, including clinical and statistical assumptions supporting any sample size calculations	5
Recruitment	#15	Strategies for achieving adequate participant enrolment to reach target sample size	6
**Methods: Assignment of interventions (for controlled trials)**			
Allocation: sequence generation	#16a	Method of generating the allocation sequence (eg, computer-generated random numbers), and list of any factors for stratification. To reduce predictability of a random sequence, details of any planned restriction (eg, blocking) should be provided in a separate document that is unavailable to those who enrol participants or assign interventions	7
Allocation concealment mechanism	#16b	Mechanism of implementing the allocation sequence (eg, central telephone; sequentially numbered, opaque,	7

For peer review only

		sealed envelopes), describing any steps to conceal the sequence until interventions are assigned	
Allocation: implementation	#16c	Who will generate the allocation sequence, who will enrol participants, and who will assign participants to interventions	7
Blinding (masking)	#17a	Who will be blinded after assignment to interventions (eg, trial participants, care providers, outcome assessors, data analysts), and how	10
Blinding (masking): emergency unblinding	#17b	If blinded, circumstances under which unblinding is permissible, and procedure for revealing a participant's allocated intervention during the trial	10
**Methods: Data collection, management, and analysis**			
Data collection plan	#18a	Plans for assessment and collection of outcome, baseline, and other trial data, including any related processes to promote data quality (eg, duplicate measurements, training of assessors) and a description of study instruments (eg, questionnaires, laboratory tests) along with their reliability and validity, if known. Reference to where data collection forms can be found, if not in the protocol	5 & 6

For peer review only

Data collection plan: retention	#18b	Plans to promote participant retention and complete follow-up, including list of any outcome data to be collected for participants who discontinue or deviate from intervention protocols	5 & 6
Data management	#19	Plans for data entry, coding, security, and storage, including any related processes to promote data quality (eg, double data entry; range checks for data values). Reference to where details of data management procedures can be found, if not in the protocol	5 & 12
Statistics: outcomes	#20a	Statistical methods for analysing primary and secondary outcomes. Reference to where other details of the statistical analysis plan can be found, if not in the protocol	11
Statistics: additional analyses	#20b	Methods for any additional analyses (eg, subgroup and adjusted analyses)	11
Statistics: analysis population and missing data	#20c	Definition of analysis population relating to protocol non-adherence (eg, as randomised analysis), and any statistical methods to handle missing data (eg, multiple imputation)	11

Methods: Monitoring

Data monitoring: formal committee	#21a	Composition of data monitoring committee (DMC); summary of its role and reporting structure; statement of whether it is independent from the sponsor and competing interests; and reference to where further	11

For peer review only

		details about its charter can be found, if not in the protocol. Alternatively, an explanation of why a DMC is not needed	
Data monitoring: interim analysis	#21b	Description of any interim analyses and stopping guidelines, including who will have access to these interim results and make the final decision to terminate the trial	N/A
Harms	#22	Plans for collecting, assessing, reporting, and managing solicited and spontaneously reported adverse events and other unintended effects of trial interventions or trial conduct	10
Auditing	#23	Frequency and procedures for auditing trial conduct, if any, and whether the process will be independent from investigators and the sponsor	12
**Ethics and dissemination**			
Research ethics approval	#24	Plans for seeking research ethics committee / institutional review board (REC / IRB) approval	12
Protocol amendments	#25	Plans for communicating important protocol modifications (eg, changes to eligibility criteria, outcomes, analyses) to relevant parties (eg, investigators, REC / IRBs, trial participants, trial registries, journals, regulators)	12

For peer review only

Consent or assent	#26a	Who will obtain informed consent or assent from potential trial participants or authorised surrogates, and how (see Item 32)	6
6Consent or assent: ancillary studies	#26b	Additional consent provisions for collection and use of participant data and biological specimens in ancillary studies, if applicable	9 & 10
Confidentiality	#27	How personal information about potential and enrolled participants will be collected, shared, and maintained in order to protect confidentiality before, during, and after the trial	12
Declaration of interests	#28	Financial and other competing interests for principal investigators for the overall trial and each study site	13
Data access	#29	Statement of who will have access to the final trial dataset, and disclosure of contractual agreements that limit such access for investigators	13
Ancillary and post trial care	#30	Provisions, if any, for ancillary and post-trial care, and for compensation to those who suffer harm from trial participation	12
Dissemination policy: trial results	#31a	Plans for investigators and sponsor to communicate trial results to participants, healthcare professionals, the public, and other relevant groups (eg, via publication, reporting in results databases, or other data sharing arrangements), including any publication restrictions	12

For peer review only

Dissemination policy: authorship	#31b	Authorship eligibility guidelines and any intended use of professional writers	12
Dissemination policy: reproducible research	#31c	Plans, if any, for granting public access to the full protocol, participant-level dataset, and statistical code	12

Appendices

Informed consent materials	#32	Model consent form and other related documentation given to participants and authorised surrogates	8 & 9
Biological specimens	#33	Plans for collection, laboratory evaluation, and storage of biological specimens for genetic or molecular analysis in the current trial and for future use in ancillary studies, if applicable	9 & 10

None The SPIRIT checklist is distributed under the terms of the Creative Commons Attribution License CC-BY-ND 3.0. This checklist can be completed online using https://www.goodreports.org/, a tool made by the EQUATOR Network in collaboration with Penelope.ai

For peer review only
